# Accuracy of odor-based microorganism identification by microbiological technologists with different years of experience: A cross-sectional study

**DOI:** 10.1016/j.heliyon.2024.e36573

**Published:** 2024-08-22

**Authors:** Tatsuya Fujihara, Hiroo Matsuo, Go Yamamoto, Atsuko Sunada, Shigeto Hamaguchi, Daisuke Onozuka, Satoshi Kutsuna

**Affiliations:** a Department of Infection Control and Prevention, Osaka University Hospital, 2-15 Yamadaoka, Suita, Osaka, 565-0871, Japan; b Department of Transformative Analysis for Human Specimen, Osaka University Graduate School of Medicine, Japan; c Department of Oral Microbe Control, Graduate School of Medicine, Osaka University, Japan

**Keywords:** Microbial identification, Odor, Accuracy, Microorganism

## Abstract

**Introduction:**

Elucidating the characteristic odors of microbes can facilitate microorganism identification. This study aimed to evaluate the accuracy of microbial identification based on odor and its association with years of experience among microbiological technologists.

**Methods:**

A cross-sectional study was conducted on February 19, 2023, in Osaka, Japan, in a laboratory capable of handling microorganisms that were rated at or below biosafety level 2. This study included 70 microbiological technologists (including 45 women) with a mean experience of 7.1 years (standard deviation, 5.7). Ten bacterial strains with distinct odors were selected. Participants were blindfolded and asked to identify the bacterial strains based on odor of cultured microbes alone. Linear and logistic regression analyses were used for data analysis. The primary outcome was the number of accurately identified bacterial strains per year of experience.

**Results:**

The number of years of experience was not significantly associated with the accuracy of odor identification (regression coefficient = 0.037 [95 % confidence interval: 0.038 to 0.113]). Additionally, generally low accuracy was noted in the identification of individual microbial species.

**Conclusions:**

Our findings indicate that microorganism identification based solely on odor is challenging. Incorporating additional information, such as visual cues, may enhance the identification accuracy.

## Highlights

• We studied odor-based identification of microbes by microbiological technologists.
• Ten bacterial strains with distinct odors were selected.
• Microorganism identification based solely on odor was challenging.
• Years of experience were not associated with odor identification accuracy.
• Incorporation of additional information, such as visual cues, may enhance accuracy.

## 1 Introduction

Mass spectrometry and nucleic acid amplification tests are used for microbial identification [1]. In addition, microbial identification can be facilitated by classical methods, such as staining, agglutination tests, and the assessment of biochemical properties, colony morphology, hemolytic activity, and characteristic odors [2]. Studies have investigated the ability of dogs to identify *Clostridioides difficile* toxin-positive stools through their sense of smell [3]. Moreover, recently, attempts have been made to identify patients with severe acute respiratory syndrome coronavirus-2 based on the odor of their sweat [4]. Strain identification based on the odor of cultured microbes can facilitate the implementation of appropriate infection control measures and antimicrobial therapy. However, the accuracy of microbiological technologists in identifying microbial strains based on their characteristic odors remains unclear. Therefore, this study aimed to determine the accuracy of microbial identification based on odor and explore its association with the years of experience among microbiological technologists. We hypothesized that more experienced microbiological technologists would demonstrate better performance in identifying microorganisms by odor alone, compared with the less experienced ones.

## 2 Materials and methods

### 2.1 Trial design and participants

For this cross-sectional single center study, we recruited microbiological technologists from the general public who had experience in microbiology laboratories, including those with concurrent roles. The exclusion criteria were as follows: presence of olfactory impairment; immunodeficiency disorders; or respiratory conditions, such as asthma or chronic obstructive pulmonary disease; and use of immunosuppressive medications. This study was conducted on February 19, 2023, in Osaka, Japan, in a laboratory capable of handling microorganisms rated at or below biosafety level 2. We collected data regarding microbial identification skills and years of experience of microbiological technologists.

### 2.2 Outcome

The primary outcome was number of identified bacterial strains per year of experience.

### 2.3 Microorganisms

For the test, we selected 10 microbial strains with distinct odors that were rated at or below biosafety level 2 [[5], [6], [7], [8], [9], [10], [11], [12], [13], [14]]. The selected microorganisms are listed in Table 1. In addition to the available selectable options, we included five other microorganisms that were commonly handled in clinical practice and were well recognized by microbiologists.

**Table 1:** Microorganisms used in the study, corresponding culture media, and associated characteristic odors.

Microorganisms	Culture media	Odor
*Haemophilus influenzae*	Chocolate agar	Mousy
*Streptococcus anginosus*	Trypticase soy agar plate with 5 % sheep blood	Caramel-like
*Staphylococcus aureus*	Egg yolk, mannitol salt agar	Cheesy
*Clostridioides difficile*	CCMA agar plate	Horse manure-like
*Bacillus subtilis*	Muller–Hinton agar	Smell chemicals from rival colonies
*Proteus mirabilis*	Trypticase soy agar plate with 5 % sheep blood	Fishy
*Pseudomonas aeruginosa*	Trypticase soy agar plate with 5 % sheep blood	Rotten potato-like
*Candida albicans*	Sabouraud agar	Yeasty
*Streptomyces species*	Trypticase soy agar plate with 5 % sheep blood	Musty
*Myroides odoratus*	Trypticase soy agar plate with 5 % sheep blood	Fruity

CCMA, combined carbon medium agar.

### 2.4 Microorganism culture conditions

Cultures for each test microorganism were prepared 2 days before the test, with the growth of the inoculated microorganisms confirmed a day before the test.

*Haemophilus influenzae* was cultured on chocolate agar for 24 h at 37 °C with 5 % CO_2_; *Streptococcus anginosus*, on 5 % sheep blood agar for 24 h at 37 °C with 5 % CO_2_; and *Staphylococcus aureus*, on mannitol salt agar with cefoxitin for 24 h at 37 °C in aerobic conditions. In addition, *C. difficile* was cultured on cycloserin cefoxitin mannitol agar for 48 h at 37 °C in anaerobic conditions, and the remaining bacteria at 37 °C in aerobic conditions for different time periods. Furthermore, *Bacillus subtilis* was cultured on 5 % sheep blood Muller–Hinton agar for 24 h, *Proteus mirabilis* on 5 % sheep blood agar for 24 h, *Pseudomonas aeruginosa* on Muller–Hinton agar for 24 h, *Candida albicans* on Sabouraud agar for 24 h, *Streptomyces* sp. on 5 % sheep blood agar for 48 h, and *Myroides* sp. on 5 % sheep blood agar for 24 h.

After an interim check on microbial growth the next day, microorganisms requiring 24 h for growth were stored at room temperature (approximately 21 °C) after 24 h. Those requiring 48 h for growth were stored at room temperature on the test day, 48 h after the initial check.

### 2.5 Study protocol

**Fig. 1:**
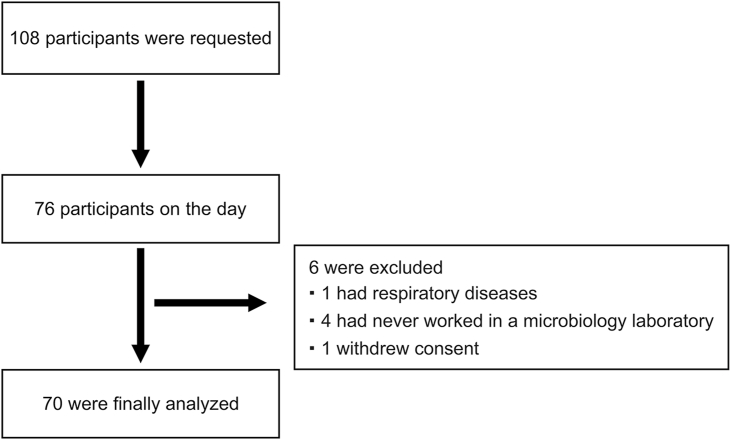
Recruitment process for participants Following public recruitment, 108 individuals who expressed interest in participating in the study are included; among them, 32 were absent on the scheduled day. Among the remaining individuals, five were excluded (four lacked experience working in a microbiology laboratory and one had a respiratory condition), whereas one withdrew consent.

The participants were blinded from the information regarding the study before the test day. On the test day, the participants were asked to wear an eye mask to prevent them from seeing the culture medium or colonies. Subsequently, they were handed the prepared culture media of the 10 microorganisms and asked to identify the bacterial strain based on the odor. The detailed study protocol and test procedures are presented in Table 2 and Fig. 1, respectively. The participants provided responses using a multiple-choice format with 15 options, with each correct identification being scored as one point (total score range: 0–10).

**Table 2:** Protocol used for the analysis of the different culture media by the participants.

Protocol	
Step 1.	The participants wore gloves (HALYARD, LAVENDER NITRILE, POWDER-FREE GLOVES) and either aprons (SARAYA Disposable pe apron) or their own lab coats.
Step 2.	The participants wore eye masks (ENN LLC: Trademark Registration Number: 6368205) to prevent them from seeing the culture media.
Step 3.	The test supervisor handed them the culture media to be used in the experiment.
Step 4.	The participants were given 10 s to smell the odor of the microbial growth on the culture media.
Step 5.	The test supervisor collected the culture media while the participants still had their eye masks on and could not see the media.
Step 6.	The participants were asked to remove their eye masks.
Step 7.	The participants were allowed 30 s to write their responses on pre-prepared answer sheets.

The protocol was as follows: Steps 2 to 7 were repeated 10 times, with one attempt for each microorganism.

### 2.6 Sample size

The target sample size was 100 participants. Assuming a significance level of 0.05, power of 0.8, three covariates, and moderate effect size (0.15), the calculated estimated sample size was 85.

### 2.7 Data collection and statistical analysis

Participants’ characteristics, such as sex and years of experience as microbiological technologists were collected using a questionnaire (Supplementary Data 1). Next, the number of correct responses for each strain was documented and the median values were calculated. For analysis of the primary outcome, linear regression was performed with the number of years of experience in a microbiology laboratory as the independent variable and the number of correct answers as the dependent variable. Furthermore, we analyzed the accuracy of identification of each microorganism using the concordance statistic (c-statistic) of the logistic regression model, which was indicated by the area under the receiver operating characteristic curve (ROC). C-statistic values > 0.7 indicated good model discrimination. All statistical analyses were performed using Stata 18.0 (Stata Corp., College Station, TX, USA). Statistical significance was set at a two-tailed P value < 0.05.

## 3 Results

**Fig. 2:**
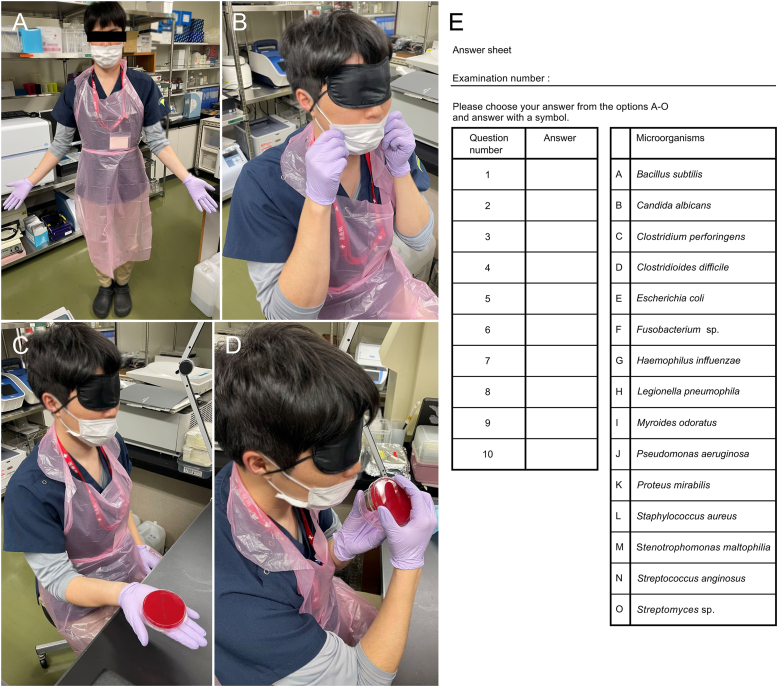
Research protocol (A) All participants wear gloves, aprons, or their own lab coats (Table 2, Step 1). (B) Participants are instructed to take their designated seats and wear a blindfold, which is confirmed by the test supervisor. If participants are wearing a mouth mask, they are also instructed to lower their mouth masks to smell the odors (Table 2, Step 2). (C) The test supervisor hands the culture media to the blinded participants (Table 2, Step 3). (D) The participants are instructed to confirm the odor of the microorganisms within 10 s. Subsequently, the test supervisor collects the culture media from the blinded participants (Table 2, Step 4, 5). (E) After the culture media is collected, the participants remove their eye masks and receive an answer sheet, on which they are asked to select one option among 15 multiple choices provided in the right column (Table 2, Step 6, 7).

One hundred eight individuals expressed interest in participating in the study; among them, 32 were absent on the scheduled day. Among the remaining individuals, five were excluded (four lacked experience working in a microbiology laboratory and one had a respiratory condition), whereas one withdrew consent. In total, 70 participants were included in the final analysis (Fig. 2).

The median number of years of experience as microbiological technologists was 5 (interquartile range [IQR]: 3–10), with minimum and maximum values of 1 and 27, respectively. Regarding the sex of the participants, there were 25 men and 45 women. Table 3 shows the distribution of the participants’ years of experience and sex.

**Table 3:** Participant characteristics and scores.

Characteristics and scores	
Median number of years of experience (interquartile range [IQR])	5 (3–10)
Sex (n)	
Male	25
Female	45
Median number of correct answers (IQR)	4 (2.25–5)

**Fig. 3:**
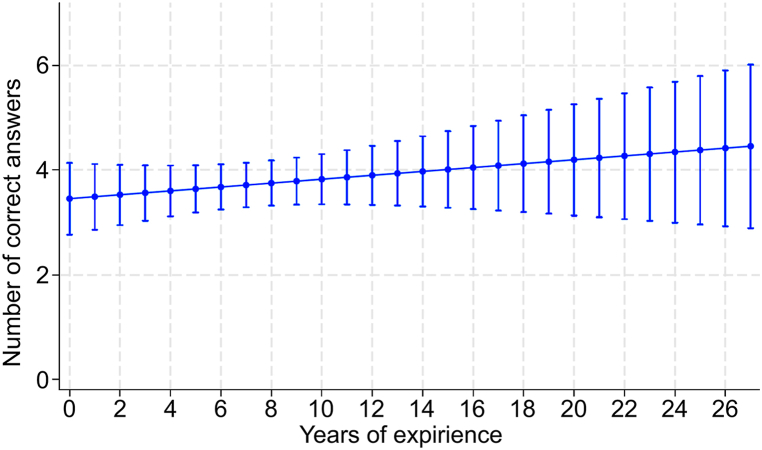
Relationship between number of years of experience of microbial testing and accurate identification of microorganisms Linear regression slopes with the “number of correct answers” as the dependent variable and “number of years of experience” as the independent variable. The vertical bars represent the 95 % confidence intervals for each category of years of experience.

**Fig. 4:**
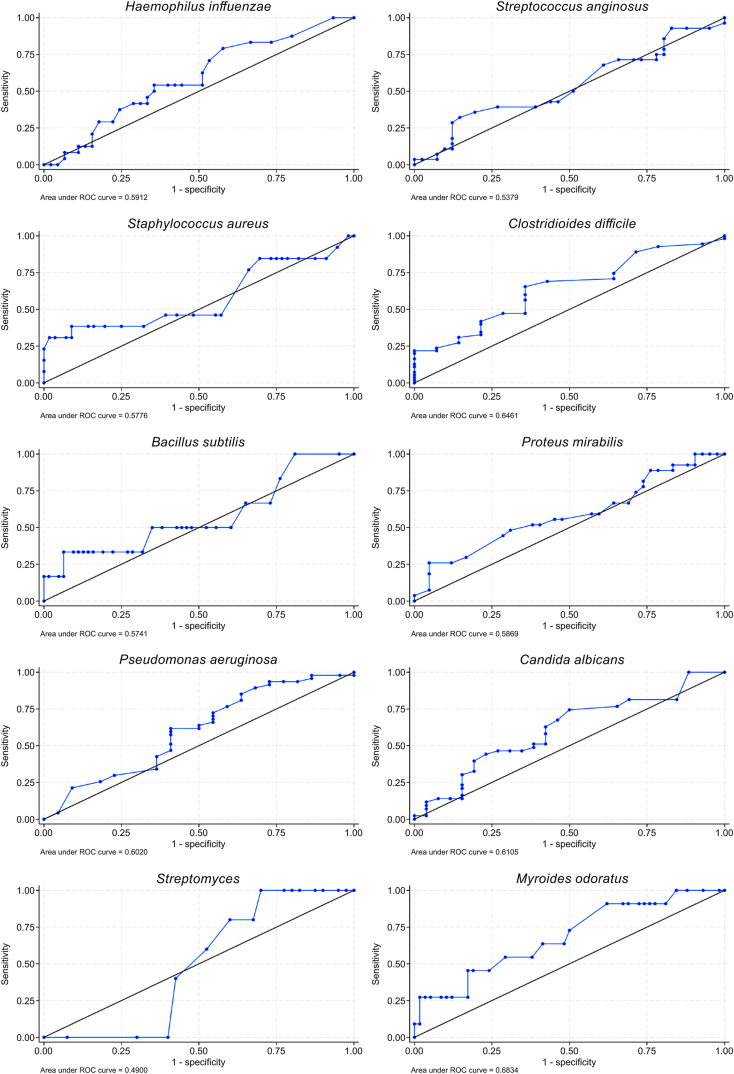
Identification accuracy for each selected microorganism The values of the area under the receiver operating characteristic curve are all <0.7, indicating modest predictive accuracy for microbial identification.

The median final test score for microorganism identification was 4 (IQR: 2.25–5) points. The number of years of experience was not significantly associated with the accuracy of odor identification (regression coefficient = 0.037; 95 % confidence interval: 0.038 to 0.113; P = 0.328; Fig. 3). Fig. 4 shows the ROC curves for the accuracy of identification of each selected strain.

## 4 Discussion

We evaluated whether the accuracy of microbial identification based solely on odor was associated with the number of years of experience of microbiological technologists. Our findings indicated that the number of years of experience was not significantly associated with the accuracy of microbial identification based on odor alone, which refuted our hypothesis. Furthermore, the accuracy of identifying individual microbial species was generally low.

Previous research has suggested that olfactory thresholds may decrease with age [15]. As experienced microbiology clinical technologists may be relatively older, improvement in the microbial identification ability due to experience may be countered by the aging-related decline in olfactory function. Moreover, recent automated methods for bacterial identification may have led to less emphasis on identification of bacterial colonies based on the odor.

A previous study showed that nurses achieved high sensitivity and specificity in identifying *C. difficile* based on olfaction [16]. However, the nurses were not blinded to the characteristics of the patients or stool samples. Moreover, the nurses could not accurately identify *C. difficile* solely based on the stool odor (sensitivity, 26 %; specificity, 69 %). This suggests that other information, including clinical symptoms and diarrhea, may have added to the diagnostic performance observed with olfaction [17]. Similarly, experienced microbiologists may incorporate additional information, including visual cues, to improve odor-based identification accuracy. Visual information has been shown to influence olfaction [18,19]. Furthermore, multiple sensory information sources, including visual cues, such as colony characteristics and growth media, are reportedly important for microbial identification [2]. Therefore, even for microbial specimens with distinct odors, the reliance on visual information before and after olfactory evaluation may have contributed to the observed low accuracy.

This study has limitations. First, we did not reach the target sample size, which may have resulted in inadequate statistical power to detect a correlation. Second, odors can be influenced by various factors, including alcohol consumption, age, menstrual cycle, environment, and race [15,[20], [21], [22]]. Third, we did not thoroughly consider other individual factors of the participants, which may have had confounding effects on the results. Fourth, the presence of olfactory impairment was based on self-reports by the participants. Therefore, inaccurate self-reports and unawareness of existing olfactory impairments may have influenced our findings. Fifth, the use of a multiple-choice response format may have increased the identification rate. Therefore, to ensure the achievement of the target sample size in future studies, it is necessary to recruit participants from a broader range, investigate several factors related to olfaction, and stratify them accordingly. Finally, olfactory discrimination can also be influenced by clinical or visual information. For example, by comparing groups that incorporate olfactory information with visual cues from microorganisms whose colony morphology is similar and those that do not, it may be possible to assess whether olfactory cues contribute to identifying microorganisms in a situation more akin to the actual testing system.

## 5 Conclusions

This study demonstrated that the accuracy of identifying microorganisms solely based on odor is generally low and that it was not associated with the number of years of experience among microbiological technologists. Our findings indicate that solely relying on odor for microorganism identification is challenging and that additional information, including visual cues, should be incorporated to enhance the accuracy of identifying microorganisms.

## Ethical statement

This study was approved by the Ethical Review Board of Osaka University Hospital (approval number: 22370) and was conducted in accordance with the Declaration of Helsinki. Written informed consent was obtained from all participants.

## Data availability statement

The datasets generated and/or analyzed during the current study are available from the corresponding author on reasonable request.

## Funding

This research did not receive any specific funding.

## CRediT authorship contribution statement

**Tatsuya Fujihara:** Writing – original draft, Methodology, Investigation. **Hiroo Matsuo:** Writing – review & editing, Methodology, Investigation. **Go Yamamoto:** Writing – review & editing, Methodology, Investigation. **Atsuko Sunada:** Methodology, Investigation. **Shigeto Hamaguchi:** Writing – review & editing, Methodology, Investigation. **Daisuke Onozuka:** Formal analysis, Data curation. **Satoshi Kutsuna:** Writing – review & editing, Methodology, Investigation, Data curation.

## Declaration of competing interest

The authors declare that they have no known competing financial interests or personal relationships that could have appeared to influence the work reported in this paper.
