# Supplementary material for: Accuracy of odor-based microorganism identification by microbiological technologists with different years of experience: A cross-sectional study
Source: Heliyon. 2024 Aug 22;10(16):e36573. doi: 10.1016/j.heliyon.2024.e36573 (PMC11385762; doi:10.1016/j.heliyon.2024.e36573)
Supplement: Multimedia component 1 [file mmc1.docx]

**Supplementary Data 1.** Preliminary questionnaire

Examination number:

| 1. How many years have you been working as a clinical laboratory technician? | Years |
| --- | --- |
| 1. How many years have you been working as a microbiology clinical laboratory technician, including any part-time positions? | Years |
| 1. What is your sex? | male・female・other |
| 1. Do you have any underlying medical conditions that require regular check-ups? | Yes・No |
| If you answered “Yes”.  4-1. Are you currently undergoing outpatient visits due to coexisting conditions of immunodeficiency, such as taking immunosuppressive medications like steroids?  4-2. Are you currently undergoing outpatient visits for respiratory conditions such as chronic obstructive pulmonary disease or bronchial asthma? | Yes・No  Yes・No |
| 1. Are you currently visiting the hospital due to a loss of sense of smell? | Yes・No |

If you answered "yes" to questions 4 and 5, we regret to inform you that you are unable to participate in this study. We apologize for any inconvenience this may cause and appreciate your understanding.
